# Low-power and area-efficient memristor based non-volatile D latch and flip-flop: Design and analysis

**DOI:** 10.1371/journal.pone.0300073

**Published:** 2024-03-07

**Authors:** Haroon Rasheed S., Rajeev Pankaj Nelapati

**Affiliations:** School of Electronics Engineering, Vellore Institute of Technology, Vellore, Tamilnadu, India; Dayalbagh Educational Institute (Deemed University), INDIA

## Abstract

In recent years, non-volatile memory elements have become highly appealing for memory applications to implement a new class of storage memory that could replace flash memories in sequential logic applications, with features such as compactness, low power, fast processing speed, high endurance, and retention. The memristor is one such non-volatile element that fits the fundamental blocks of sequential logic circuits, the latch and flip-flop; hence, in this article, a non-volatile latch architecture using memristor ratioed logic (MRL) inverter and CMOS components is focused, with an additional memristor as a memory element. A Verilog-A model was used to create the memristor element. The simulation findings validated the compact, low-voltage, and reliable design of the latch design. We evolved in technology enough to create a master-slave flip-flop and arrange it to function as a counter and a shift register. Power, number of elements, cell size, energy, programming time, and robustness are compared to comparable non-volatile topologies. The proposed non-volatile latch proves non-volatility and can store data with a 24% reduction in power consumption and a near 10% reduction in area.

## Introduction

In this big data era with explosive growth, the protection of data is crucial and challenging, as power interruptions are unpredictable. To reduce data loss, backup technology is one of the solutions that can restore the data and enhance the efficiency of the system by providing uninterrupted and stable operation. In the conventional data backup process, volatile memory data is transferred to non-volatile memory and restored after the completion of the downtime. The latency in the transfer of data depends on the memory type and architecture of the system. The way latency is reduced is crucial to enhance the efficiency of data backup.

In applications driven by low power Very Large-Scale Integrated circuits (VLSI) systems like the Internet of Things (IoT), energy efficiency is utmost important during low activity [1], which is tampered with basically due to volatile memory elements that cannot be power gated during this period in order to retain their state. The leakage of power during this period deteriorates the efficiency of the system and can be improved by introducing the NVM, which has the ability to save its current state during an inactive period without power and helps in energy savings. This leads to almost zero power consumption [2] during standby mode with system state savings and a fast wake-up transition. In addition to energy efficiency, area overhead should be improved.

Oxide based Resistive Random-Access Memories (Ox RRAM) are non-volatile in behavior, and most promising candidate of all the available Resistive RAM’s [3–5] due to their fast switching, easy fabrication, high density of integration, good compatibility with CMOS technology, large data processing, good endurance (10^6^–10^8^), and supports IoT applications [6]. The basic state holding elements in sequential circuits are the latch and the flip-flop, which, if designed with non-volatile elements, can ease backup and restore capability during power interruptions. Many circuits were proposed in the literature to achieve an optimal design that guarantees smaller delay, lower power consumption, robustness, and minimum chip area [7–22].

Memristor based sequential circuits were not investigated much. In this paper, a non-volatile latch is proposed, followed by a flip-flop, which finds its use in almost every digital circuit, like shift registers, counters, frequency dividers, and all storage elements. This paper aims to achieve reliable non-volatile low voltage design with better power, cell size, delay, and robustness.

The design uses an Ox RRAM based memristive device that can store the last processed data even if power is interrupted, taking advantage of its memory feature. This avoids the additional hardware and processing involved in traditional backup and restore mechanisms. Ox RRAM characterization data is acquired using the compact Verilog-A code of the VTEAM [23] model, which shows the non-linearity property required with an adjustable threshold voltage. Among the available memristive logic families, CMOS compatible Memristor Ratioed Logic (MRL) [24] is used.

The paper is organized as follows: Memristor and its model is presented initially, followed by architecture and design of the proposed sequential circuit, non-volatile D-latch. This novel latch design is validated in flip-flop, counter, and register designs in the next section. An improved version of the traditional CMOS latch and flip-flop is suggested in the succeeding section. Results and discussion with process, voltage, and temperature analysis is carried out in subsequent section. Finally, the conclusion of the paper is presented.

## Memristor and its model

**Fig 1:**
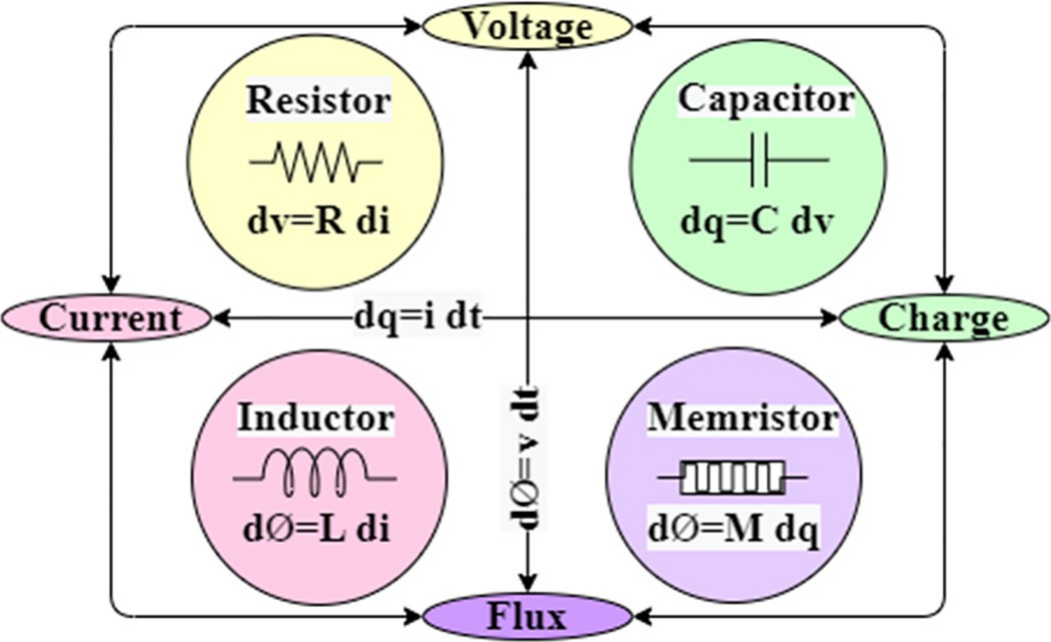
Relation between four fundamental circuit elements: Resistor, capacitor, inductor and memristor.

Memristors are two terminal passive non-volatile elements with a variable resistance theoretically conceptualized by Leon Chua in 1971 [25] that relates the missing link between flux and charge as shown in Fig 1. The variable resistance [26] depends on the amount of electric charge and the direction it has flown through a device. The memristor remembers its recent resistance state in power failure and holds it till the power is turned again. This nanoscale device was realized physically at Hewlett-Packard (HP) Labs in 2008 [27]. The identity of the memristor is its hysteresis loop, which shows non-linear behavior. Many models realize the characteristics of a memristor in terms of voltage, current, and state variable is dependent on the application.

**Fig 2:**
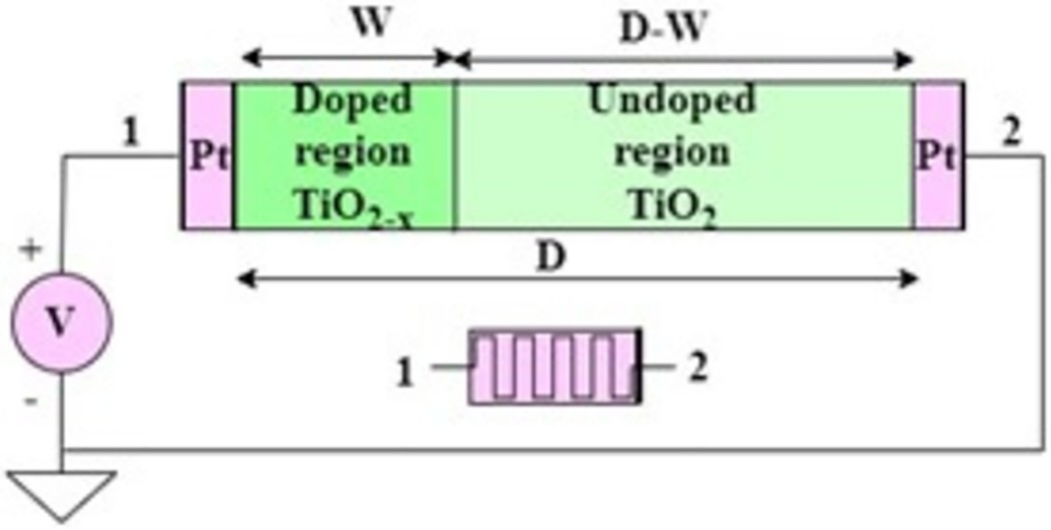
Biasing of memristor (inset—Memristor symbol).

The analytical memristor model of metal-oxide-metal architecture proposed by HP labs is given below. The relationship between flux and charge can be expressed as in Eq (1). The geometric view and symbol of the device is shown in Fig 2. The difference between the values of *R*_*OFF*_ and, *R*_*ON*_ is several orders of magnitude; this huge margin makes it suitable to store binary values and implement the non-volatility characteristic of a device.


$$M\left(q\right)=\frac{d\varphi \left(t\right)}{dq\left(t\right)}=\frac{\int v\left(t\right)dt}{\int i\left(t\right)dt}=\frac{v\left(t\right)}{i\left(t\right)}$$ (1)

$$v\left(t\right)=M\left(q\right)\cdot i\left(t\right)$$ (2)

In Eq (2), *v*(*t*) specifies the potential difference across the memristor, *i*(*t*) is the flow of current, and *M*(*q*) is the memristance that depends on the applied charge. This memristance *M*(*q*) in terms of device parameters is given by:

$$M\left(q\right)=R_{ON}\frac{w\left(t\right)}{D}+R_{OFF}\left(1-\frac{w\left(t\right)}{D}\right)$$ (3)

On substitution of Eqs (3) into (2), we get

$$v\left(t\right)=\left[R_{ON}\frac{w\left(t\right)}{D}+R_{OFF}\left(1-\frac{w\left(t\right)}{D}\right)\right]\cdot i\left(t\right)$$ (4)

where *R*_*ON*_ is the resistance at the doped region, *R*_*OFF*_ is the resistance at the undoped region, *w*(*t*) is the width of the doped region, and *D* is the device length.

The selection of memristor model is based on its accuracy, compatibility with the specific technology being used, and the requirements of the application. The VTEAM model is well established in the literature and offers sufficient accuracy compared to other previously proposed mathematical models using fitting parameters, demonstrating generality, flexibility, and computational efficiency with a certain threshold voltage. The memristor changes its state only if the applied voltage exceeds this threshold voltage. Therefore, it is useful in digital applications.

The voltage threshold adaptive memristor (VTEAM) model [23], proposed by Kvatinsky et al. in which the resistance of the memristor is changed from one value to the other with reference to the threshold voltage i.e., it has the ability to switch electrically between ON and OFF states with variable doping concentration. The resistance of the memristor varies according to the applied voltage. The device changes its state from high resistance state (HRS) to low resistance state (LRS) for a positive threshold and vice versa for a negative threshold. These resistance states define the logic states: HRS as logic 0, and LRS as logic 1. If the bias is in between these two thresholds, then the resistance state will be unaffected, i.e., previous resistance state is continued.

A voltage-controlled time-invariant memristive device, VTEAM model is described from Eqs (5) to (8). The gradient of state variable is given below in Eq (5)

$$\frac{dw}{dt}=\left\{\begin{array}{ll} k_{OFF}\;{\left(\frac{v\left(t\right)}{v_{OFF}}-1\right)}^{\alpha_{OFF}}.\;f_{OFF}\left(w\right), & 0<v_{OFF}<v\\ 0, & v_{ON}<v<v_{OFF}\\ k_{OFF}\;{\left(\frac{v\left(t\right)}{v_{OFF}}-1\right)}^{\alpha_{OFF}}.\;f_{OFF}\left(w\right), & v<v_{ON}<0 \end{array}\right.$$ (5)

$$i\left(t\right)={\left[R_{ON}+\frac{R_{OFF}-R_{ON}}{w_{OFF}-w_{ON}}\left(w-w_{ON}\right)\right]}^{-1}\cdot v\left(t\right)$$ (6)

$$i\left(t\right)=\frac{e^{\;-\;\frac{\lambda }{w_{OFF}-w_{ON}}\left(w-w_{ON}\right)}}{R_{ON}}\cdot v\left(t\right)$$ (7)

where *λ* is normalizing factor,

$$e^{\lambda }=\left(\frac{R_{OFF}}{R_{ON}}\right)$$ (8)

Here, *k*_*OFF*_, *k*_*ON*_, *α*_*OFF*_, *α*_*ON*_ are fitting constants, and *v*_*ON*_ and *v*_*OFF*_ are threshold voltages. *f*_*OFF*_(*w*) and *f*_*ON*_(*w*) defines the dependence of the derivative of the state variable on w and acts as window function which confines the window functions between w_ON_ and w_OFF_. The current–voltage (i—v) relationship is not defined intrinsically in the model and hence can be freely opted from any i—v characteristic. The parameters used for the model in the design are tabulated in Table 1.

**Table 1:** Numerical values of parameters used in the model.

Parameter	Physical Interpretation	Numerical Values
**R**_**ON**_	ON resistance	1500 Ω
**R**_**OFF**_	OFF resistance	100 KΩ
**D**	Device length	3 nm
**μ**_**V**_	Ion mobility	1e^-15^ *m*^2^/*s*.*V*
**f(w)**	Window function	0<w<1
**k**_**ON**_	Fitting constant	-10
**k**_**OFF**_	Fitting constant	5e^-4^
**α**_**ON**_	Fitting constant	3
**α**_**OFF**_	Fitting constant	1

## Proposed memristor based sequential circuits

A sequential circuit is an amalgamation of combinational logic circuit and a memory element in which output is determined based on the present and past input. The most important element which finds its application in almost all memory applications is the bi-stable circuit that has the ability to store a bit of information. Depending on the way, the bi-stable circuit responds to the input, it is categorized as a latch and a flip-flop. A circuit in which output changes at any instant of input is termed as a latch (asynchronous) and is varied only at the transition in case of a flip-flop (synchronous). This work is focused on the most popular D-latch, a more reliable and basic building block for complex memory applications.

### MRL inverter

**Fig 3:**
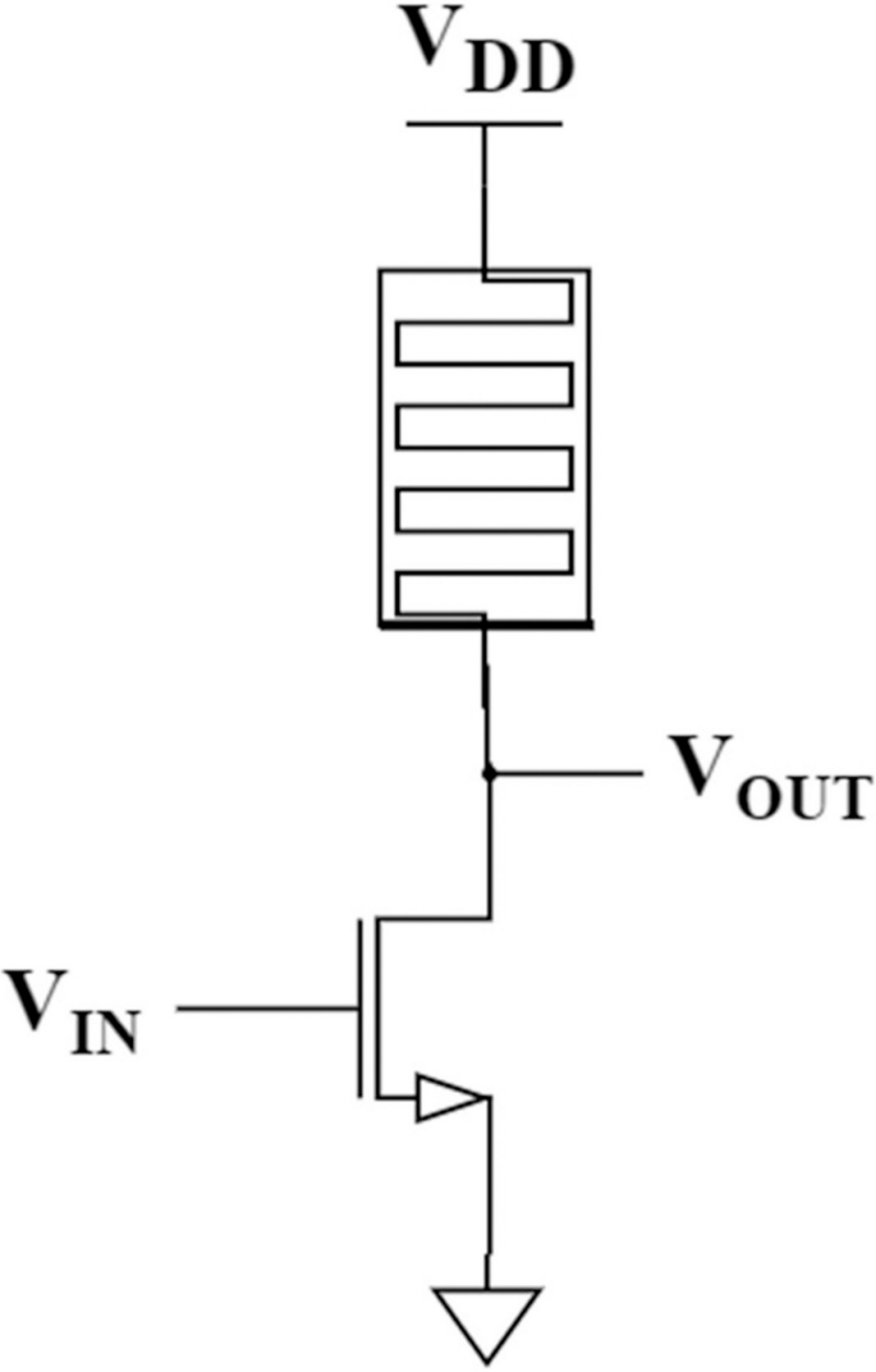
MRL Inverter.

The proposed D-latch uses a MRL inverter [28, 29] with a memristor and a NMOS transistor as a pull-up and pull-down devices respectively as in Fig 3 instead of the traditional CMOS inverter. This design reduces a significant amount of chip area, delay, and power compared to the traditional CMOS design [30] at the cost of static power due to ratioed logic. The memristor in MRL circuit is meant for logic computation and not for storing a logic state.

The inverter is biased with a dc voltage of V_DD_ at the top electrode of the memristor; input (V_IN_) is applied at the gate of the transistor. When V_IN_ = 1 (high voltage), the nmos transistor turns ON, offering a near zero resistance, i.e., *R*_*trans*_ ≃ 0, whereas the memristor is in conduction with a memristance of *R*_*M*_ = *R*_*ON*_. The output voltage, *V*_*OUT*_ can be evaluated using the potential divider rule as in Eq 9.


$$V_{OUT}=\frac{R_{trans}}{R_{ON}+R_{trans}}.V_{DD}≃0=Logic\;0$$ (9)

Similarly, when V_IN_ = 0 (low voltage) then the transistor turns OFF, offering a large resistance i.e., *R*_*trans*_ ≃ ∞ whereas the memristor is in conduction with a memristance of *R*_*M*_ = *R*_*ON*._ The output voltage, *V*_*OUT*_ is evaluated by Eq 10.


$$V_{OUT}=\frac{R_{trans}}{R_{ON}+R_{trans}}.V_{DD}≃V_{DD}=Logic\;\;1$$ (10)

### Proposed non-volatile D-latch

**Fig 4:**
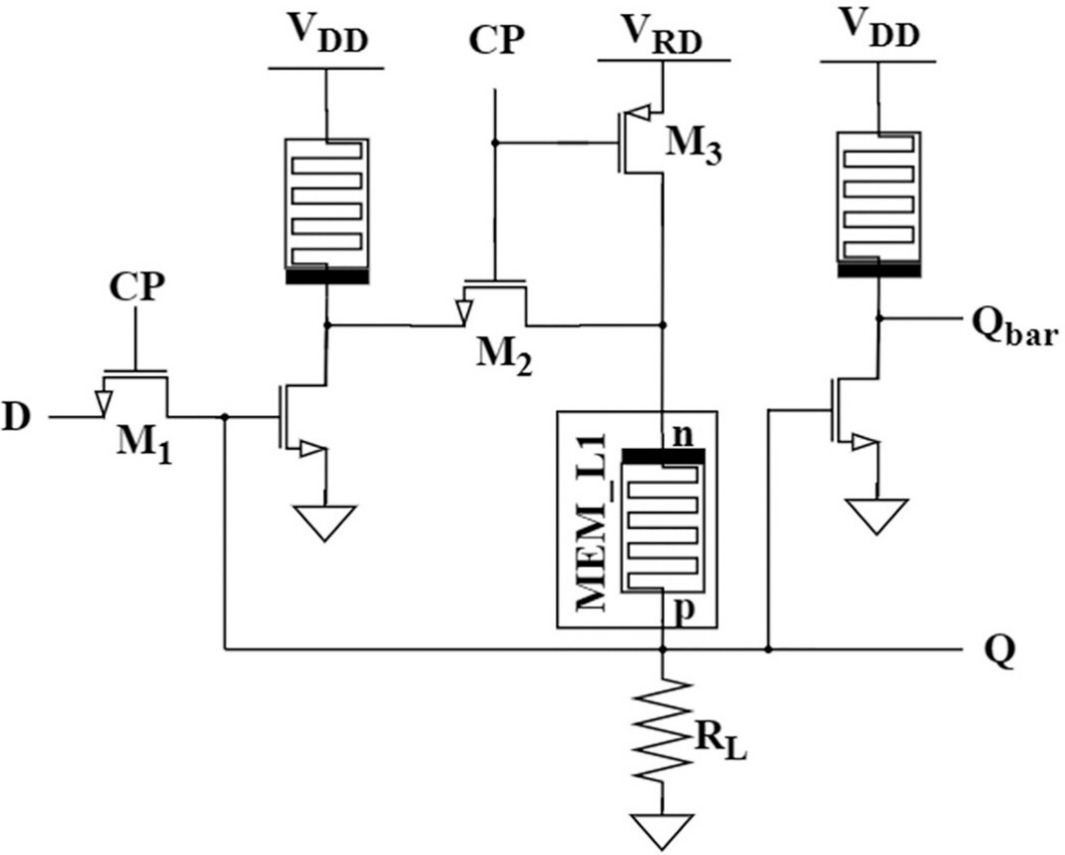
D-Latch with MRL inverters and memristor.

Fig 4 depicts the topology of non-volatile D latch with MRL inverters, CMOS components, and an additional memristor, MEM_L1. The design was previously evaluated in [7, 31]. We adapted the circuit further by using MRL inverters instead of traditional CMOS inverters and reduced the number of inverters, minimizing the area overhead of the chip.

**Fig 5:**
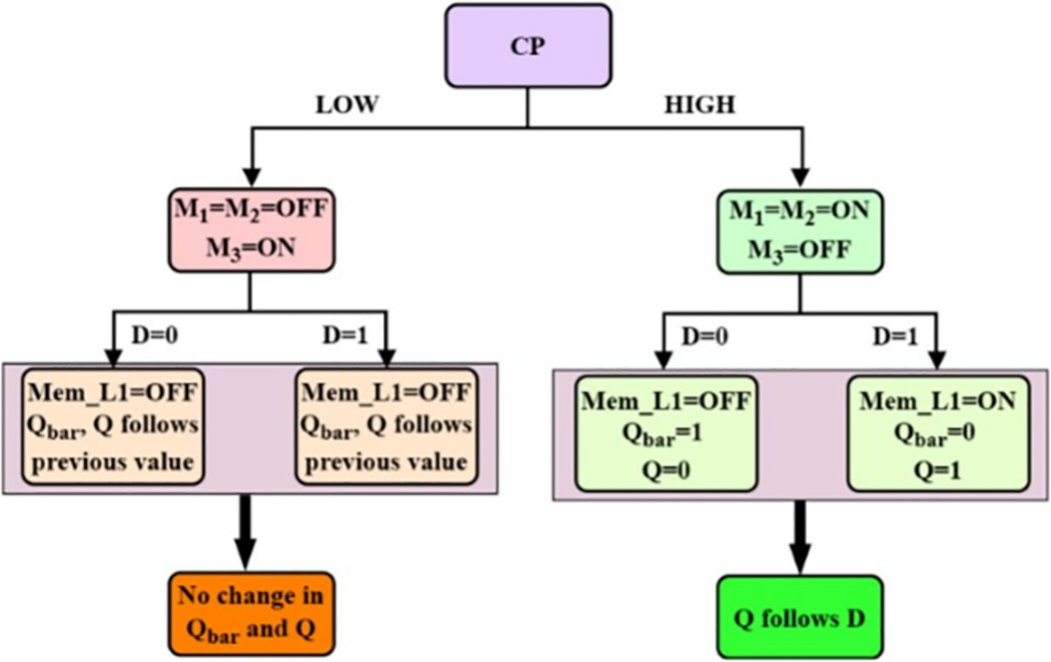
Functional flow of non-volatile D-Latch.

The proposed D latch circuit consists of three memristors, four NMOS transistors, a PMOS transistor, and a load resistor R_L_. The purpose of M_3_ along with memristor MEM_L1, and load resistor *R*_*L*_ is for reading operation. The load resistor R_L_ lies in the range of *R*_*ON*_ and *R*_*OFF*_ i.e., *R*_*ON*_ << *R*_*L*_ << *R*_*OFF*_ to ensure the output with binary values 0 and 1 based on resistance states of memristor. The functionality of the proposed D-latch is analysed in Fig 5 as follows:

- When CP = 1, the transistors M_1_ and M_2_ are ON and the M_3_ is OFF, leading to a short circuit between the input and output terminals. Due to short circuit, output, Q follows the input. This provides an advantage of reduced delay. The inverter produces the complementary output, Q_bar_.
- When CP = 0, the transistors M_1_ and M_2_ are OFF and the M_3_ is ON, isolating the input from output. The output is not influenced by the present input. Therefore, the active circuit includes transistor M_3_, memristor MEM_L1 and a load resistor R_L_ that performs the read operation. Depending on the voltage across memristor, the output state is read based on Eq (11).
$$V_{MEM\_L1}=\frac{R_{L}}{R_{trans}+R_{M}+R_{L}}.V_{RD}$$ (11)

As the transistor M_3_ is ON, the resistance offered is near zero and can be neglected. This voltage across memristor is compared with the threshold voltages to extract the output. If the voltage falls between the thresholds, V_ON_ and V_OFF_, the output follows the previous state of Q. If the voltage exceeds V_ON_, then the output produced is in high state and if the voltage goes below V_OFF_, then the output produced is in a low state.

**Fig 6:**
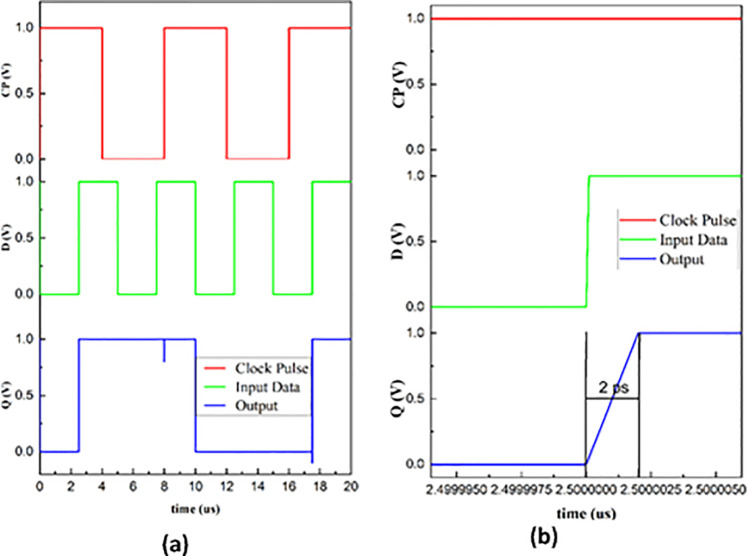
(a) Simulated waveform of MRL based non-volatile D-Latch. (b)Propagation delay in MRL based non-volatile D-Latch.

The non-volatile D-latch circuit proposed is designed with the following specifications: V_RD_ = 0.1V, V_DD_ = 1V, *R*_*L*_ = 10KΩ, and CP = 1V with a time period of 8 μs. The value of V_RD_ is selected as such because it should not override the state of the memristor during a low state of the clock pulse. It should cause the voltage across memristor V_MEM_L1_ as in Eq (11) to fall in between threshold voltages so that the state is read without change. Preferably, the value lies between the threshold voltages. As the threshold is 0.2V, the read potential is set to 0.1V. The simulated transient response of the design follows its truth table, Table 2, as exhibited in Fig 6a. The Fig 6b depicts the delay associated with the design.

**Table 2:** Truth table of D-Latch.

Clock Pulse, CP	Input, D	Output, Q
**0**	X	No change in state
**1**	0	0, Reset
**1**	1	1, Set

### Proposed master-slave D Flip-flop

**Fig 7:**
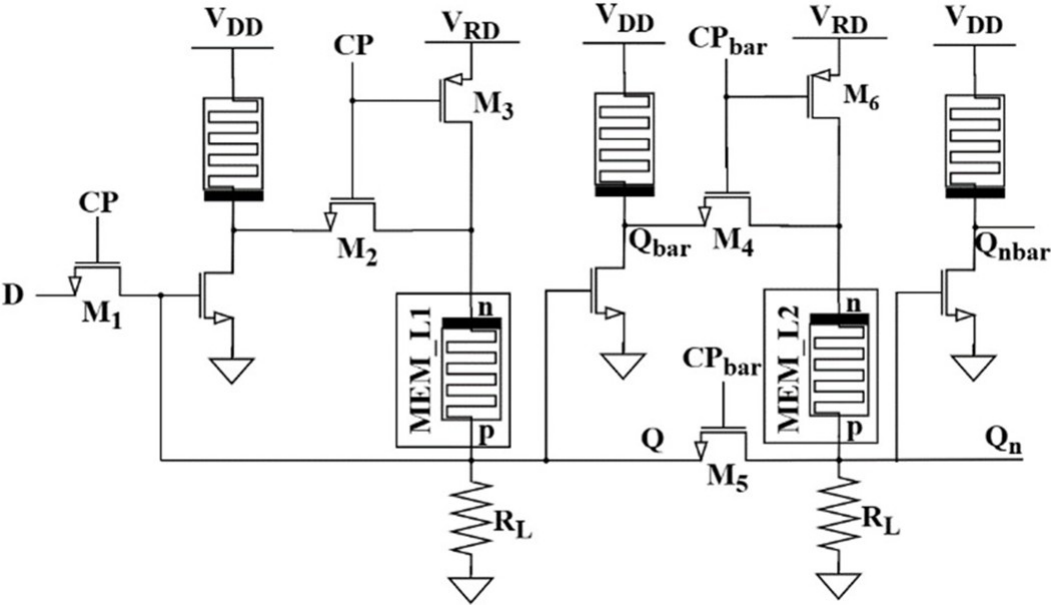
Master-slave D-flip-flop with MRL inverters and memristor.

Robinett, Warren, et al. proposed a non-volatile master slave flip-flop initially in 2010 [32]. This scheme is used to develop a more stable flip-flop independent of disturbances compared to a level triggered latch. Since the level sensitive circuit (latch) changes its output in accordance with the input changes, disturbances in the input can cause the output to change, making the circuit more sensitive to disturbances. This is why it cannot be widely used in large-scale digital integrated circuits. To address this issue, edge-triggered flip-flops are used, in which the output changes only at the rising or falling edge of the clock signal, thus making the circuit less sensitive to disturbances compared to latches. The proposed flip-flop circuit is a cascading of two proposed memristor based non-volatile latches configured as shown in Fig 7. The latch on the left acts as a master, while the one on the right acts as a slave.

During CP = 1, M_1_, M_2_, and M_6_ are ON while M_3_, M_4_, and M_5_ are OFF. The master latch at the left will be in writing mode, and the input D is latched to Q by changing the memristance of the memristor MEM_L1, which is isolated from the slave latch as it is in reading mode. The output Q_n_ depends only on the memristance of MEM_L2 but not the input D; if the memristance is *R*_*OFF*_, then Q_n_ is 0, otherwise 1. Similarly, during CP = 0, M_1_, M_2_, and M_6_ are OFF while M_3_, M_4_, and M_5_ are ON. Now the master latch enters reading mode, whereas the slave latch enables writing mode. The data of the master latch, Q, is fetched into the slave latch. If MEM_L1 presents *R*_*OFF*_, then the output Q_n_ is set to 0; otherwise, 1.

**Fig 8:**
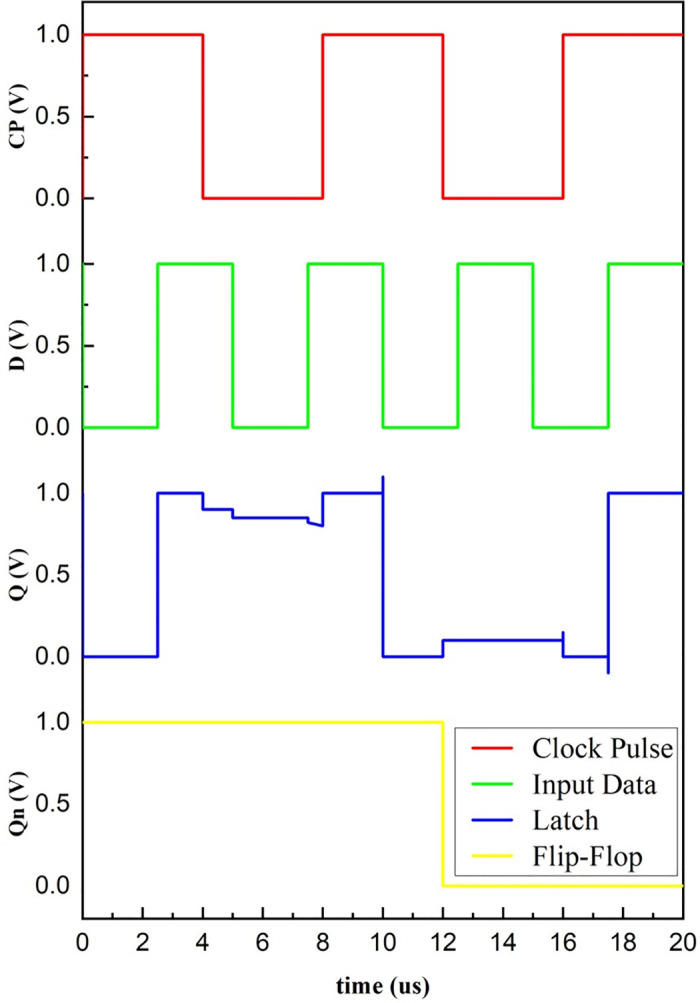
Simulated waveform of master-slave D flip-flop.

Depending on the analysis carried out, the output Q_n_ of the proposed master-slave D flip-flop changes when the clock pulse changes from high to low. In simple terms, the flip-flop responds to the falling edge of the clock pulse, whereas the state is maintained unchanged for the rest of the time with the non-volatile property. The simulated response of the proposed design is exhibited in Fig 8. It satisfies the functionality of the D flip-flop. A little output degradation is always expected in a master-slave configuration due to the loading, timing constraints like propagation delay, and clock skew. Due to these reasons, the latch output in Fig 8 is not constant.

### Proposed mod-counter and shift register

**Fig 9:**
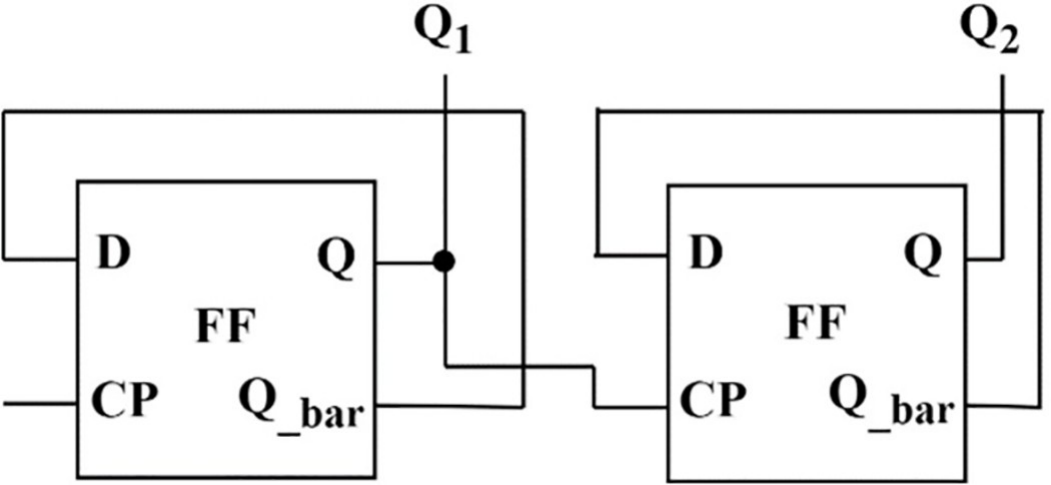
Mod-4 counter.

**Fig 10:**
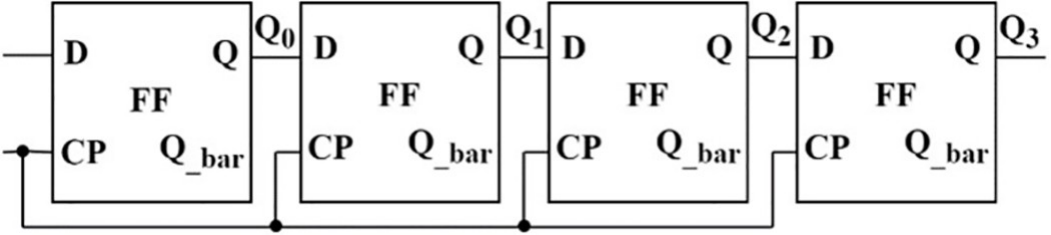
A four-bit SISO shift register.

**Fig 11:**
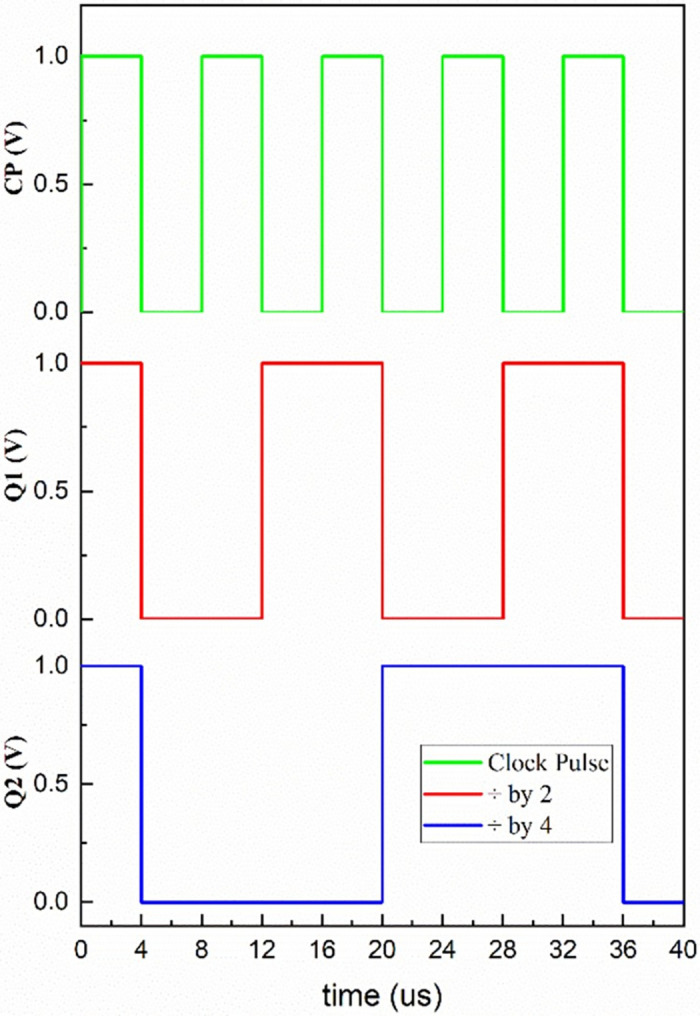
Simulated waveform Mod-4 counter.

**Fig 12:**
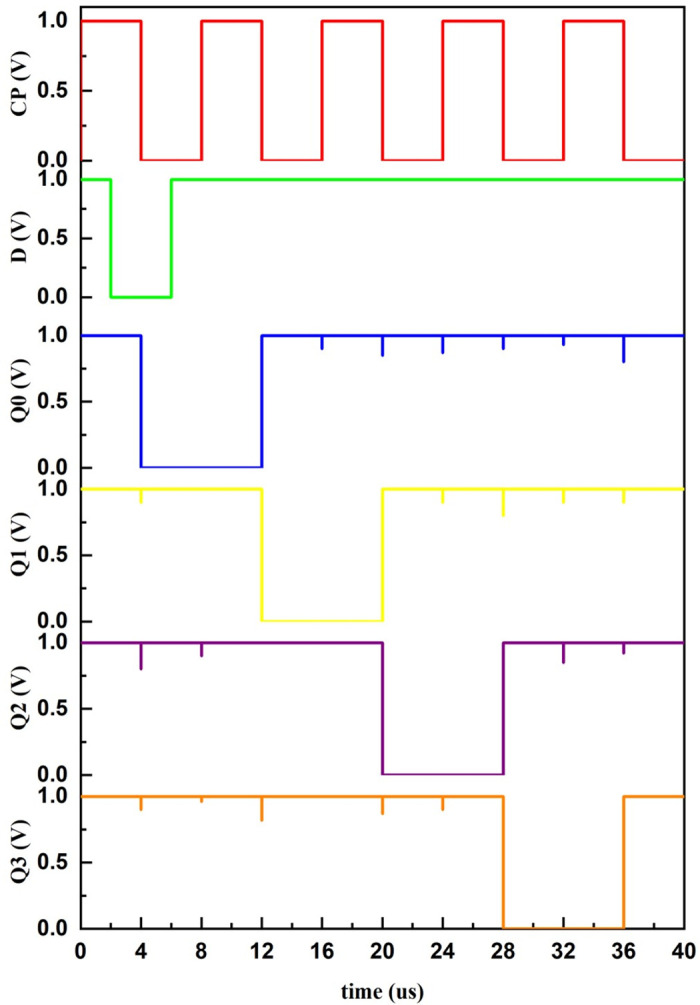
Simulated waveform of four-bit SISO register.

The flip-flop presented in Fig 7 is used to develop a few applications. One is the mod-counter as shown in Fig 9 and the four-bit serial in–serial out (SISO) shift register as shown in Fig 10, with their simulated results depicted in Figs 11 and 12, respectively. A mod-counter acts as a frequency divider circuit, and a mod-4 counter is proposed here using two master-slave flip-flops with a clock pulse input. It provides two outputs, Q_1_ and Q_2_, as shown in Fig 9, that increase the count by one for each clock pulse excitation until it reaches its maximum count of three. After approaching its maximum, it restarts from zero and continues to perform until it reaches its maximum once again.

A four-bit SISO shift register with four master-slave flip-flops connected in series with a common clock pulse signal is also simulated and verified. Based on the occurrence of each clock pulse, the input is shifted by one position. The data at the occurrence of first clock comes out of the register after four clock pulses, as in Fig 12.

## Improved design of CMOS flip-flop

**Fig 13:**
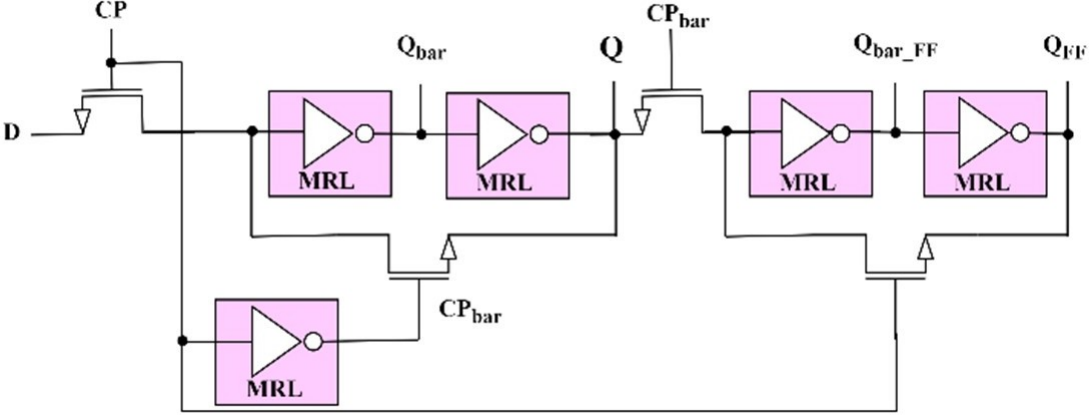
MRL inverter based traditional CMOS flip-flop.

**Fig 14:**
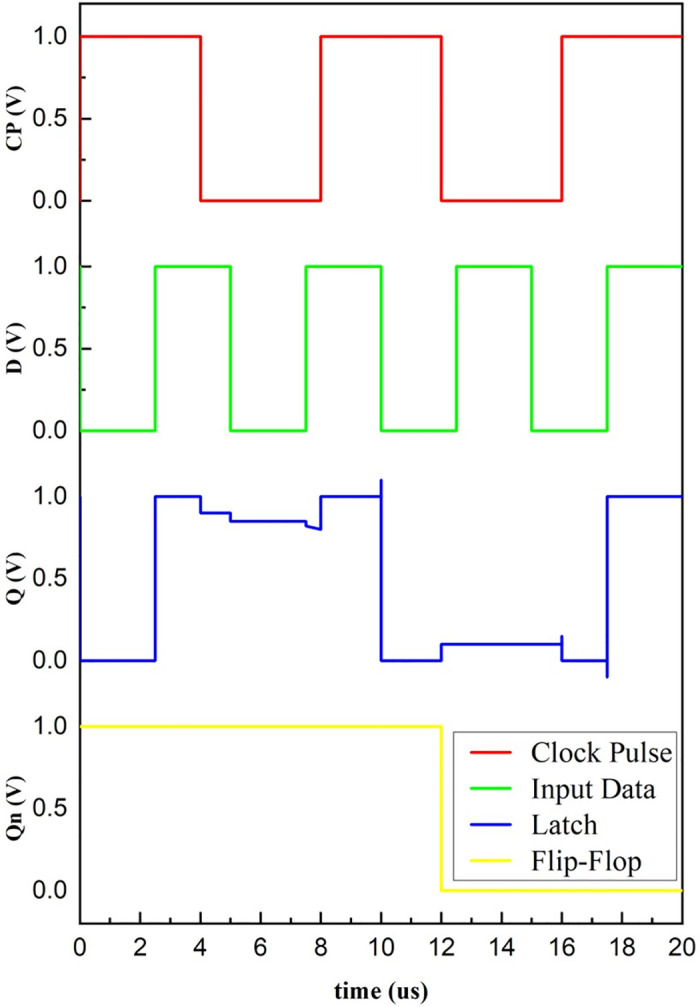
Simulated waveform of MRL inverter based traditional CMOS flip-flop.

In addition to the design of a non-volatile latch architecture, we also recommend a traditional CMOS latch and flip-flop with reduced cell size using MRL inverters as in Fig 3 in place of traditional CMOS inverters, which could reduce the area occupied on the chip as the number of transistors is reduced. An improved CMOS flip-flop circuit with MRL inverters is depicted in Fig 13. The functionality of latch and flip-flop is verified with simulations, as shown in Fig 14.

## Results and discussion

The implementation of all hybrid memristor-based circuits was carried out using the industry standard Cadence Virtuoso system design platform in 45 nm technology with a supply voltage of 1V. The transient response of various circuits is verified.

The design area of any circuit is a very important parameter, and its optimization is crucial to efficient layout design. The greater the number of components in the design, the greater the area and power consumption. A MOS transistor consumes a minimum area of 784 nm^2^, and a memristor occupies 9 nm^2^ [33], 98.85% less space compared to a MOS transistor. A transistor has a size of 6F^2^, whereas a memristor has a size of 4F^2^, where F indicates the smallest feature size in CMOS technology [34]. The comparison is made accordingly.

Power consumption in a CMOS circuit is a combination of static and dynamic power consumption. The static power consumption is due to leakage current, and the dynamic power consumption is a combination of switching power and short circuit power, as given by Eq 12.


$$P_{total}=P_{static}+P_{dynamic}$$ (12)

Delay is the time required for the circuit to respond to an input and produce an output. The average time delay is the average of the rise and fall time delays, as given by Eq (13).


$$Avg\_delay=\frac{T_{phl}+T_{plh}}{2}$$ (13)

Our MRL inverter-based non-volatile memristor-based D latch has a small size of 42 F^2^ compared to the other non-volatile topologies [7–9, 14, 15] due to the fewest number of components used, as shown in Table 3. The simulated results revealed that the non-volatile latch has better energy efficiency and more power consumption than the CMOS latch. The memristor resistive states play a role in performing the desired operation, and hence this resistive state switching needs more power consumption. For every clock cycle, the data is written and stored even in non-programmed power interrupts. The average energy per clock cycle is evaluated based on the possible clock changes. The energy consumption in storing and restoring the data is also compared, and it is inferred that the NVL design consumes less energy as it does not require additional components as in a traditional CMOS design. The restoration time is shorter as memristor remembers the recent state even during power-down.

**Table 3:** Comparison of non-volatile memristor based D-Latch.

Parameter	This work	[15] RHRNL	[35]	[14]	[9]	[8]	[7]	[14] CMOS latch
**Total no. of Components**	8	12	14	8	16	9	10	8
**No. of Transistors**	5	8	8	7	8	7	9	8
**No. of Memristors**	3	4	6	1	8	2	1	NIL
**Cell Size (F** ^**2**^ **)**	42	64	72	46	80	50	58	48
**Avg. power consumption (μW)**	21.1	73.41	29.45	27.6	N.A	N.A	N.A	15.15
**Avg. energy per clock (J)**	0.113 n	N.A	N.A	27.6 p	N.A	N.A	N.A	1.51 n
**Delay (s)**	2 p	1.29	N.A	N.A	N.A	N.A	N.A	10 n
**Time to store data (s)**	0	0	N.A	0	0	0	0	N.A

N.A-Not available

Due to the reduced count of transistors and the absence of memristors in general, the CMOS latch is proven to be the most energy-efficient and area-efficient, but with volatile behavior. The proposed MRL inverter-based non-volatile latch reduces the area to 42 F^2^ from 48 F^2^. The non-volatile latch is proved to be the best in terms of area and power than the non-volatile latches in [7–9, 14, 16]. It is better even in store and restore activities. The non-volatility provides the advantage of increasing chip density due to fewer components, a necessity in technology scale reduction. The fewer-component design proved to be a good benefit in terms of scalability and power consumption. The latch design offers reduced component design in NVL designs and has a power consumption of 21.1 μW, an average energy per clock of 0.113 nJ, and a delay of 2 ps. A reduction of power consumption by 24% and area by nearly 10% is achieved compared to the non-volatile latch designs available.

The "Clock-to-Q" and "Data-to-Q" delays are key timing characteristics for latches. These delays provide the timing details of a latch. The clock-to-Q delay is the time it takes for a signal to propagate from the clock input to the latch’s output (Q). It is important to determine whether the output data is valid after a clock edge. It affects the entire setup time and the maximum clock frequency that a circuit can attain.


$$t_{CQ}=t_{Q}-t_{Clk}=10\text{pS}$$

Data-to-Q delay is the time it takes for the latch’s output (Q) to change in response to a change in the data input (D), assuming the clock is already active. It determines the minimal amount of time that data must remain stable before the clock edge to guarantee proper latch functioning. It’s also connected to how sensitive the latch is to changes in the input data.


$$t_{DQ}=t_{Q}-t_{DATA}=2\text{pS}$$

Like any emerging technology, memristor-based latch implementation faces practical challenges like Variability and Non-Ideal behavior, Integration with CMOS Technology, efficient write and read Operations, endurance and reliability during repeated write cycles, and fabrication with consistent and reproducible properties. The potential solutions include usage of advanced calibration techniques, redundancy, and error correction codes, techniques like back-end-of-line integration and advanced fabrication processes, optimized algorithms, and write and read assist circuits.

The long-term reliability and stability of oxide-based memristor-based circuits, particularly D latches, remains a crucial challenge for practical implementation. Factors like conductivity drift, retention loss, electrochemical degradation, and circuit-level instabilities can potentially affect the long-term reliability and stability. Approaches like material process and optimization, circuit design techniques such as error correction and compensation circuits, optimizing operating voltages and currents, incorporating redundancy strategies, and cycling endurance tests can be taken into consideration to enhance reliability and stability.

The master-slave flip-flip proposed has the advantage of lower cell size compared to traditional CMOS flip-flops as well as CMOS flip-flops with MRL inverters, as shown in Table 4. The power consumed is less in a non-volatile master-slave flip-flop. This could benefit applications where area is a major constraint and low-power is a priority. The design also offers storage of data during power interruptions.

**Table 4:** Comparison of non-volatile memristor based master-slave flip-flop.

Parameter	This work	Improved CMOS Flip-flop with MRL inverters [14]	CMOS Flip-flop [14]
**Total no. of Components**	14	14	14
**No. of Transistors**	9	10	14
**No. of Memristors**	5	4	NIL
**Cell Size (F** ^**2**^ **)**	74	76	84
**Avg. power consumption (μW)**	35.1	31.6	N.A
**Time to store data (s)**	0	Volatile	Volatile

N.A-Not available

### PVT performance

The proposed latch and flip-flop performance is carried out at different process corners like FF (fast fast), SS (slow slow), TT (typical typical), FS (fast slow), and SF (slow fast) at different voltages (0.8, 0.9, 1,1.1, and 1.2V) and for temperature variation (0, 27 and 80° C). The results are the same except at 0° C for SS and SF corners. The design works better at 27 and 80° C for all the corners.

## Conclusion

In this work, we present a MRL inverter-based non-volatile latch using CMOS components and a memristor with two resistive states to store logic 0 and logic 1 data. The circuit operates at low voltage, and its functioning is thoroughly examined. A master-slave D flip-flop, Mod 2 and 4 counters, and a four-bit shift register were shown using the same. At different programming voltages, a rigorous comparison with alternative non-volatile latch topologies is performed in terms of area, power, delay, reliability, and robustness. The memristor and MRL inverter-based latch have been shown to have non-volatile features and the ability to store data with a 24% reduction in power and a near 10% reduction in area. The manufacturing community will almost probably be building better sequential logic circuits influenced by the revolutionary non-volatile latches in the near future.
