# Supplementary material for: Low-power and area-efficient memristor based non-volatile D latch and flip-flop: Design and analysis
Source: PLoS One. 2024 Mar 7;19(3):e0300073. doi: 10.1371/journal.pone.0300073 (PMC10919681; doi:10.1371/journal.pone.0300073)
Supplement: Attachment — Submitted filename: Response to Reviewers.docx [file pone.0300073.s001.docx]

**Reviewer #1:**

The paper titled "Low-power and area-efficient memristor based non-volatile D latch and flip-flop: design and analysis", presents a novel design of a low-power, area-efficient non-volatile D latch and flip-flop using memristor ratioed logic (MRL) inverters and CMOS components. The design uses a Verilog-A model for simulating the memristor element, focusing on achieving compactness and energy efficiency. The paper is well-structured, addresses a significant problem, and provides a novel solution. However, some revisions are recommended to improve the clarity and impact of the paper before publication.
Major Comments:
1. While the paper compares the proposed design with similar and other non-volatile technologies, a more detailed analysis, including specific performance metrics and benchmarks, would strengthen the argument. In this regard adding following paper would be useful:
a. Memristor-based Nonvolatile Synchronous Flip-flop Circuits
b. Reliable, high-performance, and nonvolatile hybrid SRAM/MRAM-based structures for reconfigurable nanoscale logic devices
c. Low Power, and Highly Reliable Single Event Upset Immune Latch for Nanoscale CMOS Technologies
d. Realization of Memristor based D-Latch
e. A Task-Schedulable Nonvolatile Spintronic Field-Programmable Gate Array
f. A Novel Design for Low Power Re-RAM Based Non-volatile Flip Flop Using
g. High-Performance Radiation-Hardened Spintronic Retention Latch and Flip-Flop for Highly Reliable Processors
h. High-performance and soft error immune spintronic retention latch for highly reliable processors.

The comparison focuses primarily on oxide-based ReRAM. The references have been included in the introduction part of the manuscript on non-volatile latches and flip-flops. Among the oxide-based ReRAM references, a, d, and f, inclusion is prioritised based on available data.

2. Insights into the practical implementation challenges and potential solutions would make the paper more applicable to real-world scenarios.

Like any emerging technology, memristor-based latch implementation faces practical challenges. Here are some insights into these challenges and potential solutions:

a. Variability and Non-Ideal Behaviour:

Challenge: Memristors may exhibit variability in their characteristics due to manufacturing processes and material variations. Non-ideal behaviour, such as asymmetry and drift, can also be challenging.

Solution: Advanced calibration techniques, redundancy, and error correction codes can help mitigate variability and compensate for non-ideal behaviour. Adaptive algorithms may be employed to adjust for changes in memristor characteristics over time.

b. Integration with CMOS Technology:

Challenge: Integrating memristors with existing CMOS technology poses challenges in terms of compatibility, interface circuitry, and ensuring reliable operation.

Solution: Research focuses on developing hybrid CMOS-memristor circuits and optimized interfaces. Techniques like back-end-of-line integration and advanced fabrication processes help address integration challenges.

c. Write and Read Operations:

Challenge: Achieving reliable and efficient write and read operations, especially in large-scale memristor arrays, can be challenging due to sneak paths, disturbances, and non-linear behaviour.

Solution: Use of optimized algorithms, error correction mechanisms, and carefully designed read and write circuits. Techniques like write and read assist circuits can enhance reliability.

d. Endurance and Reliability:

Challenge: Memristors may have limited endurance, and repeated write cycles can degrade their performance.

Solution: Implement wear-levelling techniques, error correction codes, and advanced algorithms to distribute write and read operations evenly across the memristor array, prolonging the device's lifespan.

e. Fabrication Challenges:

Challenge: Fabricating memristors with consistent and reproducible properties on a large scale can be challenging.

Solution: Ongoing research focuses on improving fabrication techniques, materials, and processes to enhance reproducibility and reliability.

The shorten practical challenges and solutions in design have been added in Results and discussion section at line 277.

3. A more detailed explanation of the chosen memristor model and its suitability for the proposed design would be beneficial.

This has been appended in the manuscript’s Memristor and its model from line 96.

4. The paper could include a brief discussion on the expected long-term reliability and stability of the employed memristor and proposed design.

Oxide based memristors have emerged as promising candidates for building low-power, high-density logic circuits due to their non-volatile memory, analog computing capabilities, and compatibility with CMOS technology. However, ensuring the long-term reliability and stability of memristor-based circuits, particularly D latches, remains a crucial challenge for practical implementation.

Several factors can potentially affect the long-term reliability and stability of oxide based memristor-based D latches:

a. Conductivity Drift: Over time, the resistance states of the oxide based memristors might gradually drift due to factors like oxygen migration, electrode oxidation, or trapped charges. This drift can lead to misinterpretation of the stored logic state and malfunctioning of the latch.
b. Retention Loss: Leakage currents within the memristor or across the insulating layer can gradually erase the stored information. This loss of retention capability weakens the memory function of the latch and ultimately leads to data corruption.
c. Electrochemical Degradation: Repeated write/read operations can induce electrochemical reactions within the oxide layer or at the electrode interfaces. These reactions can cause material degradation, leading to changes in memristor properties and potentially affecting circuit functionality.
d. Circuit-Level Instabilities: Interactions between the memristors and other circuit components, such as parasitic resistances or crosstalk, can introduce instabilities in the overall latch operation. These instabilities can manifest as noise, glitches, or unpredictable behaviour.

Strategies for Improving Reliability and Stability:

Several approaches can be taken to mitigate these degradation mechanisms and enhance the long-term reliability and stability of oxide based memristor-based D latches:

a. Material and process optimization: Developing purer oxide materials, optimizing electrode materials and interfaces, and refining fabrication processes can minimize defects and improve memristor uniformity, leading to better stability.
b. Circuit design techniques: Employing error correction and compensation circuits, optimizing operating voltages and currents, and incorporating redundancy strategies can address drift, leakage, and circuit-level instabilities.
c. Cycling endurance tests: Performing accelerated aging tests and analysing post-test memristor characteristics can provide valuable insights into potential degradation mechanisms and guide further optimization efforts.

The shorten presentation of long-term reliability and stability of memristor and proposed design has been included in Results and discussion section from line 283.

Minor Comments
1. Ensure consistency in the use of technical terms throughout the paper. For example, if you use "non-volatile D latch" in one section, avoid switching to "D-latch non-volatile" in another.
2. Pay attention to subject-verb agreement, especially in complex sentences that involve technical jargon. For instance, "The latch and flip-flop designs is..." should be "The latch and flip-flop designs are..."

This has been updated in the manuscript wherever required.

Reviewer #2:

1. In equation (10) why the results is equal to 1. Do you mean logical one? The numerical result will be VDD.

Yes. $V_{OUT}=\frac{R_{trans}}{R_{ON}+R_{trans}}.V_{DD}\simeq1$ specifies logic 1$\simeq V_{DD}$. It has been updated in the revised manuscript in line 147.

2. To have more accurate equations for proposed inverter, you should use the real equations of MOS and memristor instead of modeling transistor with R=0 or R=infinity.

In the MRL inverter, the memristor is always ON, offering a resistance of R_ON_, whereas the nmos transistor works either in cutoff or saturation regions. The ideal resistance of a transistor is considered 0 (very small) and infinite (very large) for the cutoff and saturation regions, respectively for easy understanding.

The below analysis gives the exact analysis using accurate equations of memristor and MOS transistor.

When V_in_=0V, as V_gs_<V_t_, the nmos transistor is in the cut-off region and opposes the flow of current completely, hence offering a very high resistance, ideally infinite.

When V_in_ = V_DD_, nmos is in saturation and conducts with a near-zero resistance, offering a current of

$$I_{ds}=\frac{1}{2}\mu_{n}C_{ox}\frac{W}{L}\left( V_{gs}-V_{t} \right)^{2}$$

$$R_{trans}=\frac{V_{ds}}{I_{ds}}=\frac{V_{ds}}{\frac{1}{2}\mu_{n}C_{ox}\frac{W}{L}\left( V_{gs}-V_{t} \right)^{2}}$$

In saturation region, $V_{ds}\geq V_{gs}-V_{t}$

$$R_{trans}=\frac{2L}{\mu_{n}C_{ox}W\left( V_{gs}-V_{t} \right)}$$

From the characteristic equation of memristor shown in Fig (2),

$$R_{ON}=\frac{WD}{\mu q(t)}$$

Substitution of $R_{trans}$ and $R_{ON}$ in $V_{OUT}$ gives an exact expression for output as in the below equation.

$$V_{OUT}=\frac{\frac{2L}{\mu_{n}C_{ox}W\left( V_{gs}-V_{t} \right)}}{\left( \frac{WD}{\mu q(t)} \right)_{Mem}+\frac{2L}{\mu_{n}C_{ox}W\left( V_{gs}-V_{t} \right)}}.V_{DD}$$

This expression gives the exact V_OUT_ when applied voltage is V_DD_.

3. What is the effect of mismatch in the proposed D-latch?

Mismatch during fabrication in the proposed D-latch leads to various effects on the performance and reliability. The following is the list:

a. It varies the resistance states of memristor that corresponds to different logic levels. This can result in inconsistent behaviour and complicate the reliable storage of information.
b. Read and write operations are affected as the switching characteristic is varied due to mismatch. This may lead to difficulties in accurately reading or writing information, potentially causing errors in the stored data.
c. Variations in power consumption within the latch may happen. Some memristors may require more energy for state transitions than others, leading to uneven power distribution.
d. Timing variations between different paths within the latch. This can impact the timing margins of the latch and may lead to performance degradation or even latch failures. Mismatch can affect the setup and hold times of the latch, potentially leading to violations of these timing constraints. If the mismatch causes one path to be faster than the other, it may result in setup time violations, while a slower path might lead to hold time violations.
e. Over time, as a result of aging or environmental factors, mismatch-related effects may become more pronounced, potentially leading to reliability issues.
4. Please show the related waveforms for D-latch which can show the delay of proposed design. What is the maximum operating frequency?

It has been included in the revised manuscript with Fig 6b at line 177. The maximum operating frequency for the proposed design is 625KHz.

Reviewer #3: we have some questions:

1. What is the area of a memristors? Area is the major concern if it is high.

The area corresponds to the physical size of the memristor. As mentioned in the Results and Discussions section, the minimum area of a transistor is 784 nm^2^, and a memristor occupies only 9 nm^2^ [25], 98.85% less space compared to a MOS transistor. Single device size does not directly impact performance, but it can be relevant for factors like manufacturing yield, packing density, and thermal management. This small device reduces power consumption. Their unique electrical properties like memristance, switching speed, and endurance are more important for their functionality and applications compared to their area.

2. Why the average power of the proposed work is high in table 4.?

The average power of the master-slave flip-flop listed in Table 4 is compared with the traditional CMOS flip-flop, which is a superior one in terms of power consumption. The entry in the second column specifies the average power consumption of a traditional CMOS flip-flop with MRL inverters instead of CMOS inverters to reduce the overall cross-sectional area on a chip.

3. Why the PVT results are not shown in terms of figure?

[As the mathematical model of memristors may not always accurately represent the behavior of physical memristors, and the results obtained from such models may not be reliable](https://www.intechopen.com/chapters/59395). However, the result is included for reference in the below figure with fast-fast, fast-slow, typical-typical, slow-fast and slow-slow process corners with V_DD_ of 0.8V, 1V and 1.2V at temperatures of 0,27, and 80⁰ C.

Fig : PVT analysis of memristor based non-volatile D latch.

4. Why the latch output is not constant in figure 8?

A little output degradation is always expected in a master-slave configuration due to the loading, timing constraints like propagation delay, and clock skew. Due to these reasons, the latch output in Fig. 8 is not constant.

5. Why the author did not talked about setup and hold time of the flip-flop?

The omission was unintentional. The setup and hold time analysis is not carried out as the device is behaving ideal and the circuit depends on the mathematical model whose practical availability is still under research. The literature that has been carried out does not include this aspect. Memristor-based latches and flip-flops have been the subject of intense research, but they still face a number of hurdles and limitations. Researchers studying memristor-based circuits often concentrate on their properties. One probable reason for the lack of specific analysis of setup and hold time in the literature that has been carried out is the inherent variations in their operating principles when compared to traditional CMOS-based circuits. Memristor-based circuits may not always conform to the same timing constraints or have different requirements for reliable operation. This is the reason why it has not been performed.

6. Author should discuss the clock to q delay and data to q delay.

The "Clock-to-Q" delay and "Data-to-Q" delay are key timing characteristics for latches. These delays provide the timing details of a latch.

The Clock-to-Q delay is the time it takes for a signal to propagate from the clock input to the output (Q) of the latch. It is important to determine whether the output data is valid after a clock edge. It affects the entire setup time and the maximum clock frequency that a circuit can attain.

$t_{CQ}=t_{Q}-t_{Clk}$=10pS

Data-to-Q delay is the time it takes for the latch's output (Q) to change in response to a change in the data input (D), assuming the clock is already active. It determines the minimal amount of time that data must remain stable before the clock edge to guarantee proper latch functioning. It's also connected to how sensitive the latch is to changes in the input data.

$t_{DQ}=t_{Q}-t_{DATA}$=2pS

This has been included in the revised manuscript in Results and Discussion section from line 266.
