# Supplementary material for: Low-power and area-efficient memristor based non-volatile D latch and flip-flop: Design and analysis
Source: PLoS One. 2024 Mar 7;19(3):e0300073. doi: 10.1371/journal.pone.0300073 (PMC10919681; doi:10.1371/journal.pone.0300073)
Supplement: Peer review history

# Peer review: Decision Letter 0

**DOI:** 10.1371/journal.pone.0300073.r001

**Authors:** Gufran Ahmad

2 Jan 2024

PONE-D-23-41957Low-power and area-efficient memristor based non-volatile D latch and flip-flop: design and analysisPLOS ONE

Dear Dr. NELAPATI,

Thank you for submitting your manuscript to PLOS ONE. After careful consideration, we feel that the work is timely, and the manuscript has merit but does not fully meet PLOS ONE’s publication criteria as it currently stands. Therefore, we invite you to submit a revised version of the manuscript that could address the points raised by all the reviewers. The manuscript requires a major revision prior to a final decision.

Please submit your revised manuscript by Feb 16 2024 11:59PM. If you will need more time than this to complete your revisions, please reply to this message or contact the journal office at plosone@plos.org. When you're ready to submit your revision, log on to https://www.editorialmanager.com/pone/ and select the 'Submissions Needing Revision' folder to locate your manuscript file.

Please include the following items when submitting your revised manuscript:

- A rebuttal letter that responds to each point raised by the academic editor and reviewer(s). You should upload this letter as a separate file labeled 'Response to Reviewers'.
- A marked-up copy of your manuscript that highlights changes made to the original version. You should upload this as a separate file labeled 'Revised Manuscript with Track Changes'.
- An unmarked version of your revised paper without tracked changes. You should upload this as a separate file labeled 'Manuscript'.

If you would like to make changes to your financial disclosure, please include your updated statement in your cover letter. Guidelines for resubmitting your figure files are available below the reviewer comments at the end of this letter.

If applicable, we recommend that you deposit your laboratory protocols in protocols.io to enhance the reproducibility of your results. Protocols.io assigns your protocol its own identifier (DOI) so that it can be cited independently in the future. For instructions see: https://journals.plos.org/plosone/s/submission-guidelines#loc-laboratory-protocols. Additionally, PLOS ONE offers an option for publishing peer-reviewed Lab Protocol articles, which describe protocols hosted on protocols.io. Read more information on sharing protocols at https://plos.org/protocols?utm_medium=editorial-email&utm_source=authorletters&utm_campaign=protocols.

We look forward to receiving your revised manuscript.

Kind regards,

Gufran Ahmad

Academic Editor

PLOS ONE

Journal Requirements:

When submitting your revision, we need you to address these additional requirements.

1. Please ensure that your manuscript meets PLOS ONE's style requirements, including those for file naming. The PLOS ONE style templates can be found at 

https://journals.plos.org/plosone/s/file?id=wjVg/PLOSOne_formatting_sample_main_body.pdf and 

https://journals.plos.org/plosone/s/file?id=ba62/PLOSOne_formatting_sample_title_authors_affiliations.pdf

2. Please note that PLOS ONE has specific guidelines on code sharing for submissions in which author-generated code underpins the findings in the manuscript. In these cases, all author-generated code must be made available without restrictions upon publication of the work. Please review our guidelines at https://journals.plos.org/plosone/s/materials-and-software-sharing#loc-sharing-code and ensure that your code is shared in a way that follows best practice and facilitates reproducibility and reuse.

3. We note that your Data Availability Statement is currently as follows: All relevant data are within the manuscript and its Supporting Information files.

Please confirm at this time whether or not your submission contains all raw data required to replicate the results of your study. Authors must share the “minimal data set” for their submission. PLOS defines the minimal data set to consist of the data required to replicate all study findings reported in the article, as well as related metadata and methods (https://journals.plos.org/plosone/s/data-availability#loc-minimal-data-set-definition).

For example, authors should submit the following data:

- The values behind the means, standard deviations and other measures reported;

- The values used to build graphs;

- The points extracted from images for analysis.

Authors do not need to submit their entire data set if only a portion of the data was used in the reported study.

If your submission does not contain these data, please either upload them as Supporting Information files or deposit them to a stable, public repository and provide us with the relevant URLs, DOIs, or accession numbers. For a list of recommended repositories, please see https://journals.plos.org/plosone/s/recommended-repositories.

If there are ethical or legal restrictions on sharing a de-identified data set, please explain them in detail (e.g., data contain potentially sensitive information, data are owned by a third-party organization, etc.) and who has imposed them (e.g., an ethics committee). Please also provide contact information for a data access committee, ethics committee, or other institutional body to which data requests may be sent. If data are owned by a third party, please indicate how others may request data access.

Additional Editor Comments:

Dear Dr. Nelapati,

Thank you for submitting your manuscript to PLOS ONE. After careful consideration, we feel that the work is timely, and the manuscript has merit but does not fully meet PLOS ONE’s publication criteria as it currently stands. Therefore, we invite you to submit a revised version of the manuscript that could address the points raised by all the reviewers. The manuscript requires a major revision prior to a final decision.

[Note: HTML markup is below. Please do not edit.]

Reviewers' comments:

Reviewer's Responses to Questions

**Comments to the Author**

1. Is the manuscript technically sound, and do the data support the conclusions?

The manuscript must describe a technically sound piece of scientific research with data that supports the conclusions. Experiments must have been conducted rigorously, with appropriate controls, replication, and sample sizes. The conclusions must be drawn appropriately based on the data presented. 

Reviewer #1: Yes

Reviewer #2: Yes

Reviewer #3: Yes

**********

2. Has the statistical analysis been performed appropriately and rigorously? 

Reviewer #1: Yes

Reviewer #2: Yes

Reviewer #3: No

**********

3. Have the authors made all data underlying the findings in their manuscript fully available?

The PLOS Data policy requires authors to make all data underlying the findings described in their manuscript fully available without restriction, with rare exception (please refer to the Data Availability Statement in the manuscript PDF file). The data should be provided as part of the manuscript or its supporting information, or deposited to a public repository. For example, in addition to summary statistics, the data points behind means, medians and variance measures should be available. If there are restrictions on publicly sharing data—e.g. participant privacy or use of data from a third party—those must be specified.

Reviewer #1: Yes

Reviewer #2: No

Reviewer #3: Yes

**********

4. Is the manuscript presented in an intelligible fashion and written in standard English?

PLOS ONE does not copyedit accepted manuscripts, so the language in submitted articles must be clear, correct, and unambiguous. Any typographical or grammatical errors should be corrected at revision, so please note any specific errors here.

Reviewer #1: Yes

Reviewer #2: Yes

Reviewer #3: Yes

**********

5. Review Comments to the Author

Please use the space provided to explain your answers to the questions above. You may also include additional comments for the author, including concerns about dual publication, research ethics, or publication ethics. (Please upload your review as an attachment if it exceeds 20,000 characters)

Reviewer #1: The paper titled "Low-power and area-efficient memristor based non-volatile D latch and flip-flop: design and analysis", presents a novel design of a low-power, area-efficient non-volatile D latch and flip-flop using memristor ratioed logic (MRL) inverters and CMOS components. The design uses a Verilog-A model for simulating the memristor element, focusing on achieving compactness and energy efficiency. The paper is well-structured, addresses a significant problem, and provides a novel solution. However, some revisions are recommended to improve the clarity and impact of the paper before publication.

Major Comments:

1. While the paper compares the proposed design with similar and other non-volatile technologies, a more detailed analysis, including specific performance metrics and benchmarks, would strengthen the argument. In this regard adding following paper would be useful:

a. Memristor-based Nonvolatile Synchronous Flip-flop Circuits

b. Reliable, high-performance, and nonvolatile hybrid SRAM/MRAM-based structures for reconfigurable nanoscale logic devices

c. Low Power, and Highly Reliable Single Event Upset Immune Latch for Nanoscale CMOS Technologies

d. Realization of Memristor based D-Latch

e. A Task-Schedulable Nonvolatile Spintronic Field-Programmable Gate Array

f. A Novel Design for Low Power Re-RAM Based Non-volatile Flip Flop Using

g. High-Performance Radiation-Hardened Spintronic Retention Latch and Flip-Flop for Highly Reliable Processors

h. High-performance and soft error immune spintronic retention latch for highly reliable processors

2. Insights into the practical implementation challenges and potential solutions would make the paper more applicable to real-world scenarios.

3. A more detailed explanation of the chosen memristor model and its suitability for the proposed design would be beneficial.

4. The paper could include a brief discussion on the expected long-term reliability and stability of the employed memristor and proposed design.

Minor Comments

1. Ensure consistency in the use of technical terms throughout the paper. For example, if you use "non-volatile D latch" in one section, avoid switching to "D-latch non-volatile" in another.

2. Pay attention to subject-verb agreement, especially in complex sentences that involve technical jargon. For instance, "The latch and flip-flop designs is..." should be "The latch and flip-flop designs are..."

Reviewer #2: 1. In equation (10) why the results is equal to 1. Do you mean logical one? The numerical result will be VDD.

2. To have more accurate equations for proposed inverter, you should use the real equations of MOS and memristor instead of modeling transistor with R=0 or R=infinity.

3. What is the effect of mismatch in the proposed D-latch?

4. Please show the related waveforms for D-latch which can show the delay of proposed design. What is the maximum operating frequency?

Reviewer #3: we have some quesions:

1. What is the area of a memristors?. Area is the major concern if it is high.

2. Why the average power of the proposed work is high in table 4.?

3. Why the PVT results are not shown in terms of figure?

4.Why the latch output is not constant in figure 8?

5. Why the author did not talked about setup and hold time of the flip-flp?

6. Author should discuss the clock to q delay and data to q delay.

**********

6. PLOS authors have the option to publish the peer review history of their article (what does this mean?). If published, this will include your full peer review and any attached files.

If you choose “no”, your identity will remain anonymous but your review may still be made public.

**Do you want your identity to be public for this peer review?** For information about this choice, including consent withdrawal, please see our Privacy Policy.

Reviewer #1: No

Reviewer #2: No

Reviewer #3: No

**********

[NOTE: If reviewer comments were submitted as an attachment file, they will be attached to this email and accessible via the submission site. Please log into your account, locate the manuscript record, and check for the action link "View Attachments". If this link does not appear, there are no attachment files.]

While revising your submission, please upload your figure files to the Preflight Analysis and Conversion Engine (PACE) digital diagnostic tool, https://pacev2.apexcovantage.com/. PACE helps ensure that figures meet PLOS requirements. To use PACE, you must first register as a user. Registration is free. Then, login and navigate to the UPLOAD tab, where you will find detailed instructions on how to use the tool. If you encounter any issues or have any questions when using PACE, please email PLOS at figures@plos.org. Please note that Supporting Information files do not need this step.

# Author comment: Author response to Decision Letter 0

**DOI:** 10.1371/journal.pone.0300073.r002

13 Jan 2024

We would like to thank all three Reviewers for their valuable suggests that enhanced the quality of our manuscript. we answered to all questions, some answers requires figures and equations to clarify. 

Reviewer #1: 

The paper titled "Low-power and area-efficient memristor based non-volatile D latch and flip-flop: design and analysis", presents a novel design of a low-power, area-efficient non-volatile D latch and flip-flop using memristor ratioed logic (MRL) inverters and CMOS components. The design uses a Verilog-A model for simulating the memristor element, focusing on achieving compactness and energy efficiency. The paper is well-structured, addresses a significant problem, and provides a novel solution. However, some revisions are recommended to improve the clarity and impact of the paper before publication.

Major Comments:

1. While the paper compares the proposed design with similar and other non-volatile technologies, a more detailed analysis, including specific performance metrics and benchmarks, would strengthen the argument. In this regard adding following paper would be useful:

a. Memristor-based Nonvolatile Synchronous Flip-flop Circuits

b. Reliable, high-performance, and nonvolatile hybrid SRAM/MRAM-based structures for reconfigurable nanoscale logic devices

c. Low Power, and Highly Reliable Single Event Upset Immune Latch for Nanoscale CMOS Technologies

d. Realization of Memristor based D-Latch

e. A Task-Schedulable Nonvolatile Spintronic Field-Programmable Gate Array

f. A Novel Design for Low Power Re-RAM Based Non-volatile Flip Flop Using

g. High-Performance Radiation-Hardened Spintronic Retention Latch and Flip-Flop for Highly Reliable Processors

h. High-performance and soft error immune spintronic retention latch for highly reliable processors.

The comparison focuses primarily on oxide-based ReRAM. The references have been included in the introduction part of the manuscript on non-volatile latches and flip-flops. Among the oxide-based ReRAM references, a, d, and f, inclusion is prioritised based on available data.

2. Insights into the practical implementation challenges and potential solutions would make the paper more applicable to real-world scenarios.

Like any emerging technology, memristor-based latch implementation faces practical challenges. Here are some insights into these challenges and potential solutions:

 Variability and Non-Ideal Behaviour:

Challenge: Memristors may exhibit variability in their characteristics due to manufacturing processes and material variations. Non-ideal behaviour, such as asymmetry and drift, can also be challenging.

Solution: Advanced calibration techniques, redundancy, and error correction codes can help mitigate variability and compensate for non-ideal behaviour. Adaptive algorithms may be employed to adjust for changes in memristor characteristics over time.

 Integration with CMOS Technology:

Challenge: Integrating memristors with existing CMOS technology poses challenges in terms of compatibility, interface circuitry, and ensuring reliable operation.

Solution: Research focuses on developing hybrid CMOS-memristor circuits and optimized interfaces. Techniques like back-end-of-line integration and advanced fabrication processes help address integration challenges.

 Write and Read Operations:

Challenge: Achieving reliable and efficient write and read operations, especially in large-scale memristor arrays, can be challenging due to sneak paths, disturbances, and non-linear behaviour.

Solution: Use of optimized algorithms, error correction mechanisms, and carefully designed read and write circuits. Techniques like write and read assist circuits can enhance reliability.

 Endurance and Reliability:

Challenge: Memristors may have limited endurance, and repeated write cycles can degrade their performance.

Solution: Implement wear-levelling techniques, error correction codes, and advanced algorithms to distribute write and read operations evenly across the memristor array, prolonging the device's lifespan.

 Fabrication Challenges:

Challenge: Fabricating memristors with consistent and reproducible properties on a large scale can be challenging.

Solution: Ongoing research focuses on improving fabrication techniques, materials, and processes to enhance reproducibility and reliability.

The shorten practical challenges and solutions in design have been added in Results and discussion section at line 277. 

3. A more detailed explanation of the chosen memristor model and its suitability for the proposed design would be beneficial.

This has been appended in the manuscript’s Memristor and its model from line 96.

4. The paper could include a brief discussion on the expected long-term reliability and stability of the employed memristor and proposed design.

Oxide based memristors have emerged as promising candidates for building low-power, high-density logic circuits due to their non-volatile memory, analog computing capabilities, and compatibility with CMOS technology. However, ensuring the long-term reliability and stability of memristor-based circuits, particularly D latches, remains a crucial challenge for practical implementation.

Several factors can potentially affect the long-term reliability and stability of oxide based memristor-based D latches:

 Conductivity Drift: Over time, the resistance states of the oxide based memristors might gradually drift due to factors like oxygen migration, electrode oxidation, or trapped charges. This drift can lead to misinterpretation of the stored logic state and malfunctioning of the latch.

 Retention Loss: Leakage currents within the memristor or across the insulating layer can gradually erase the stored information. This loss of retention capability weakens the memory function of the latch and ultimately leads to data corruption.

 Electrochemical Degradation: Repeated write/read operations can induce electrochemical reactions within the oxide layer or at the electrode interfaces. These reactions can cause material degradation, leading to changes in memristor properties and potentially affecting circuit functionality.

 Circuit-Level Instabilities: Interactions between the memristors and other circuit components, such as parasitic resistances or crosstalk, can introduce instabilities in the overall latch operation. These instabilities can manifest as noise, glitches, or unpredictable behaviour.

Strategies for Improving Reliability and Stability:

Several approaches can be taken to mitigate these degradation mechanisms and enhance the long-term reliability and stability of oxide based memristor-based D latches:

 Material and process optimization: Developing purer oxide materials, optimizing electrode materials and interfaces, and refining fabrication processes can minimize defects and improve memristor uniformity, leading to better stability.

 Circuit design techniques: Employing error correction and compensation circuits, optimizing operating voltages and currents, and incorporating redundancy strategies can address drift, leakage, and circuit-level instabilities.

 Cycling endurance tests: Performing accelerated aging tests and analysing post-test memristor characteristics can provide valuable insights into potential degradation mechanisms and guide further optimization efforts.

The shorten presentation of long-term reliability and stability of memristor and proposed design has been included in Results and discussion section from line 283. 

Minor Comments

1. Ensure consistency in the use of technical terms throughout the paper. For example, if you use "non-volatile D latch" in one section, avoid switching to "D-latch non-volatile" in another.

2. Pay attention to subject-verb agreement, especially in complex sentences that involve technical jargon. For instance, "The latch and flip-flop designs is..." should be "The latch and flip-flop designs are..."

This has been updated in the manuscript wherever required.

Reviewer #2: 

 In equation (10) why the results is equal to 1. Do you mean logical one? The numerical result will be VDD.

Yes. V_OUT=R_trans/(R_ON+R_trans ).V_DD≃1 specifies logic 1≃V_DD. It has been updated in the revised manuscript in line 147.

 To have more accurate equations for proposed inverter, you should use the real equations of MOS and memristor instead of modeling transistor with R=0 or R=infinity.

In the MRL inverter, the memristor is always ON, offering a resistance of RON, whereas the nmos transistor works either in cutoff or saturation regions. The ideal resistance of a transistor is considered 0 (very small) and infinite (very large) for the cutoff and saturation regions, respectively for easy understanding.

The below analysis gives the exact analysis using accurate equations of memristor and MOS transistor.

When Vin=0V, as Vgs<Vt, the nmos transistor is in the cut-off region and opposes the flow of current completely, hence offering a very high resistance, ideally infinite.

When Vin = VDD, nmos is in saturation and conducts with a near-zero resistance, offering a current of 

I_ds=1/2 μ_n C_ox W/L (V_gs-V_t )^2

R_trans=V_ds/I_ds =V_ds/(1/2 μ_n C_ox W/L (V_gs-V_t )^2 )

In saturation region, V_ds≥V_gs-V_t

R_trans=2L/(μ_n C_ox W(V_gs-V_t ) )

From the characteristic equation of memristor shown in Fig (2),

R_ON=WD/(μq(t))

Substitution of R_trans and R_ON in V_OUT gives an exact expression for output as in the below equation.

V_OUT=(2L/(μ_n C_ox W(V_gs-V_t ) ))/((WD/(μq(t)))_Mem+2L/(μ_n C_ox W(V_gs-V_t ) )).V_DD

This expression gives the exact VOUT when applied voltage is VDD.

 What is the effect of mismatch in the proposed D-latch?

Mismatch during fabrication in the proposed D-latch leads to various effects on the performance and reliability. The following is the list:

 It varies the resistance states of memristor that corresponds to different logic levels. This can result in inconsistent behaviour and complicate the reliable storage of information. 

 Read and write operations are affected as the switching characteristic is varied due to mismatch. This may lead to difficulties in accurately reading or writing information, potentially causing errors in the stored data. 

 Variations in power consumption within the latch may happen. Some memristors may require more energy for state transitions than others, leading to uneven power distribution. 

 Timing variations between different paths within the latch. This can impact the timing margins of the latch and may lead to performance degradation or even latch failures. Mismatch can affect the setup and hold times of the latch, potentially leading to violations of these timing constraints. If the mismatch causes one path to be faster than the other, it may result in setup time violations, while a slower path might lead to hold time violations.

 Over time, as a result of aging or environmental factors, mismatch-related effects may become more pronounced, potentially leading to reliability issues.

 Please show the related waveforms for D-latch which can show the delay of proposed design. What is the maximum operating frequency? 

It has been included in the revised manuscript with Fig 6b at line 177. The maximum operating frequency for the proposed design is 625KHz.

Reviewer #3: we have some questions:

1. What is the area of a memristors? Area is the major concern if it is high.

The area corresponds to the physical size of the memristor. As mentioned in the Results and Discussions section, the minimum area of a transistor is 784 nm2, and a memristor occupies only 9 nm2 [25], 98.85% less space compared to a MOS transistor. Single device size does not directly impact performance, but it can be relevant for factors like manufacturing yield, packing density, and thermal management. This small device reduces power consumption. Their unique electrical properties like memristance, switching speed, and endurance are more important for their functionality and applications compared to their area.

2. Why the average power of the proposed work is high in table 4.?

The average power of the master-slave flip-flop listed in Table 4 is compared with the traditional CMOS flip-flop, which is a superior one in terms of power consumption. The entry in the second column specifies the average power consumption of a traditional CMOS flip-flop with MRL inverters instead of CMOS inverters to reduce the overall cross-sectional area on a chip. 

3. Why the PVT results are not shown in terms of figure?

As the mathematical model of memristors may not always accurately represent the behavior of physical memristors, and the results obtained from such models may not be reliable. However, the result is included for reference in the below figure with fast-fast, fast-slow, typical-typical, slow-fast and slow-slow process corners with VDD of 0.8V, 1V and 1.2V at temperatures of 0,27, and 80⁰ C.

Fig : PVT analysis of memristor based non-volatile D latch.

4. Why the latch output is not constant in figure 8?

A little output degradation is always expected in a master-slave configuration due to the loading, timing constraints like propagation delay, and clock skew. Due to these reasons, the latch output in Fig. 8 is not constant.

5. Why the author did not talked about setup and hold time of the flip-flop?

The omission was unintentional. The setup and hold time analysis is not carried out as the device is behaving ideal and the circuit depends on the mathematical model whose practical availability is still under research. The literature that has been carried out does not include this aspect. Memristor-based latches and flip-flops have been the subject of intense research, but they still face a number of hurdles and limitations. Researchers studying memristor-based circuits often concentrate on their properties. One probable reason for the lack of specific analysis of setup and hold time in the literature that has been carried out is the inherent variations in their operating principles when compared to traditional CMOS-based circuits. Memristor-based circuits may not always conform to the same timing constraints or have different requirements for reliable operation. This is the reason why it has not been performed.

6. Author should discuss the clock to q delay and data to q delay.

The "Clock-to-Q" delay and "Data-to-Q" delay are key timing characteristics for latches. These delays provide the timing details of a latch.

The Clock-to-Q delay is the time it takes for a signal to propagate from the clock input to the output (Q) of the latch. It is important to determine whether the output data is valid after a clock edge. It affects the entire setup time and the maximum clock frequency that a circuit can attain.

t_CQ=t_Q-t_Clk=10pS

Data-to-Q delay is the time it takes for the latch's output (Q) to change in response to a change in the data input (D), assuming the clock is already active. It determines the minimal amount of time that data must remain stable before the clock edge to guarantee proper latch functioning. It's also connected to how sensitive the latch is to changes in the input data.

t_DQ=t_Q-t_DATA=2pS

This has been included in the revised manuscript in Results and Discussion section from line 266.

Attachment Submitted filename: Response to Reviewers.docx

# Peer review: Decision Letter 1

**DOI:** 10.1371/journal.pone.0300073.r003

**Authors:** Gufran Ahmad

22 Feb 2024

Low-power and area-efficient memristor based non-volatile D latch and flip-flop: design and analysis

PONE-D-23-41957R1

Dear Dr. Nelapati,

We’re pleased to inform you that your manuscript has been judged scientifically suitable for publication and will be formally accepted for publication once it meets all outstanding technical requirements.

Within one week, you’ll receive an e-mail detailing the required amendments. When these have been addressed, you’ll receive a formal acceptance letter and your manuscript will be scheduled for publication.

An invoice for payment will follow shortly after the formal acceptance. To ensure an efficient process, please log into Editorial Manager at http://www.editorialmanager.com/pone/, click the 'Update My Information' link at the top of the page, and double check that your user information is up-to-date. If you have any billing related questions, please contact our Author Billing department directly at authorbilling@plos.org.

If your institution or institutions have a press office, please notify them about your upcoming paper to help maximize its impact. If they’ll be preparing press materials, please inform our press team as soon as possible -- no later than 48 hours after receiving the formal acceptance. Your manuscript will remain under strict press embargo until 2 pm Eastern Time on the date of publication. For more information, please contact onepress@plos.org.

Kind regards,

Dr.Gufran Ahmad

Academic Editor

PLOS ONE

Additional Editor Comments

Dear Dr. Nelapati,

Thank you for submitting your manuscript to PLOS ONE. After careful evaluation and based on the respondents comments, we feel that the work has been improved. Therefore, the paper has been conditionally accepted, but changes are required. Include the results, mathematical equations, and all the necessary text in the main manuscript that you have submitted against the reviewer's inquiry.

Reviewers' comments:

Reviewer's Responses to Questions

**Comments to the Author**

1. If the authors have adequately addressed your comments raised in a previous round of review and you feel that this manuscript is now acceptable for publication, you may indicate that here to bypass the “Comments to the Author” section, enter your conflict of interest statement in the “Confidential to Editor” section, and submit your "Accept" recommendation.

Reviewer #1: All comments have been addressed

**********

2. Is the manuscript technically sound, and do the data support the conclusions?

The manuscript must describe a technically sound piece of scientific research with data that supports the conclusions. Experiments must have been conducted rigorously, with appropriate controls, replication, and sample sizes. The conclusions must be drawn appropriately based on the data presented. 

Reviewer #1: Yes

**********

3. Has the statistical analysis been performed appropriately and rigorously? 

Reviewer #1: Yes

**********

4. Have the authors made all data underlying the findings in their manuscript fully available?

The PLOS Data policy requires authors to make all data underlying the findings described in their manuscript fully available without restriction, with rare exception (please refer to the Data Availability Statement in the manuscript PDF file). The data should be provided as part of the manuscript or its supporting information, or deposited to a public repository. For example, in addition to summary statistics, the data points behind means, medians and variance measures should be available. If there are restrictions on publicly sharing data—e.g. participant privacy or use of data from a third party—those must be specified.

Reviewer #1: Yes

**********

5. Is the manuscript presented in an intelligible fashion and written in standard English?

PLOS ONE does not copyedit accepted manuscripts, so the language in submitted articles must be clear, correct, and unambiguous. Any typographical or grammatical errors should be corrected at revision, so please note any specific errors here.

Reviewer #1: Yes

**********

6. Review Comments to the Author

Please use the space provided to explain your answers to the questions above. You may also include additional comments for the author, including concerns about dual publication, research ethics, or publication ethics. (Please upload your review as an attachment if it exceeds 20,000 characters)

Reviewer #1: The authors addressed all of my previous comments and concerns. Now I can recommended the paper for publication. Thanks for your nice paper.

**********

7. PLOS authors have the option to publish the peer review history of their article (what does this mean?). If published, this will include your full peer review and any attached files.

If you choose “no”, your identity will remain anonymous but your review may still be made public.

**Do you want your identity to be public for this peer review?** For information about this choice, including consent withdrawal, please see our Privacy Policy.

Reviewer #1: No

**********

# Editor report: Acceptance letter

**DOI:** 10.1371/journal.pone.0300073.r004

**Authors:** Gufran Ahmad

26 Feb 2024

PONE-D-23-41957R1 

PLOS ONE

Dear Dr. Nelapati, 

I'm pleased to inform you that your manuscript has been deemed suitable for publication in PLOS ONE. Congratulations! Your manuscript is now being handed over to our production team.

At this stage, our production department will prepare your paper for publication. This includes ensuring the following:

* All references, tables, and figures are properly cited

* All relevant supporting information is included in the manuscript submission,

* There are no issues that prevent the paper from being properly typeset

If revisions are needed, the production department will contact you directly to resolve them. If no revisions are needed, you will receive an email when the publication date has been set. At this time, we do not offer pre-publication proofs to authors during production of the accepted work. Please keep in mind that we are working through a large volume of accepted articles, so please give us a few weeks to review your paper and let you know the next and final steps. 

Lastly, if your institution or institutions have a press office, please let them know about your upcoming paper now to help maximize its impact. If they'll be preparing press materials, please inform our press team within the next 48 hours. Your manuscript will remain under strict press embargo until 2 pm Eastern Time on the date of publication. For more information, please contact onepress@plos.org.

If we can help with anything else, please email us at customercare@plos.org.

Thank you for submitting your work to PLOS ONE and supporting open access. 

Kind regards, 

PLOS ONE Editorial Office Staff

on behalf of

Dr. Gufran Ahmad 

Academic Editor

PLOS ONE
